# *Nascer no Brasil*: the presence of a companion favors the use of best practices in delivery care in the South region of Brazil

**DOI:** 10.11606/S1518-8787.2018052006258

**Published:** 2018-01-16

**Authors:** Juliana Jacques da Costa Monguilhott, Odaléa Maria Brüggemann, Paulo Fontoura Freitas, Eleonora d'Orsi

**Affiliations:** I Instituto Federal de Educação, Ciência e Tecnologia de Santa Catarina. Departamento Acadêmico de Saúde e Serviços. Florianópolis, SC, Brasil; II Universidade Federal de Santa Catarina. Programa de Pós-Graduação em Enfermagem. Florianópolis, SC, Brasil; III Universidade do Sul de Santa Catarina. Núcleo de Orientação em Epidemiologia. Curso de Medicina. Palhoça, SC, Brasil; IV Universidade Federal de Santa Catarina. Departamento de Saúde Pública. Programa de Pós-Graduação em Saúde Coletiva. Florianópolis, SC, Brasil

**Keywords:** Humanizing Delivery, Humanization of Assistance, Patient Rights, Evidence-Based Practice, Maternal-Child Health Services, Parto Humanizado, Humanização da Assistência, Direitos do Paciente, Prática Clínica Baseada em Evidências, Serviços de Saúde Materno-Infantil

## Abstract

**OBJECTIVE:**

To analyze if the presence of a companion favors the use of best practices in the delivery care in the South region of Brazil.

**METHODS:**

This is a cross-sectional analysis of the longitudinal study *Nascer no Brasil.* We analyzed data from 2,070 women from the South region of Brazil who went into labor. The data were collected between February and August 2011, by interviews and medical records. We performed a bivariate and multivariate analysis, calculating the crude and adjusted prevalence ratios using Poisson regression with robust variance estimation. The level of significance adopted was 5%.

**RESULTS:**

Most women had a companion during labor (51.7%), but few remained during delivery (39.4%) or cesarean section (34.8%). Less than half of the women had access to several recommended practices, while non-recommended practices continue to be performed. In the model adjusted for age, education level, source of payment for the delivery, parity, and score of the Brazilian Association of Market Research Institutes, the presence of a companion was statistically associated with a greater supply of liquids and food (aPR = 1.34), dietary prescription (aPR = 1.34), use of non-pharmacological methods for pain relief (aPR = 1.37), amniotomy (aPR = 1.10), epidural or spinal analgesia (aPR = 1.84), adoption of non-lithotomy position in the delivery (aPR = 1.77), stay in the same room during labor, delivery, and postpartum (aPR = 1.62), skin-to-skin contact in the delivery (aPR = 1.81) and cesarean section (PR = 2.43), as well as reduced use of the Kristeller maneuver (aPR = 0.67), trichotomy (aPR = 0.59), and enema (aPR = 0.49).

**CONCLUSIONS:**

In the South region of Brazil, most women do not have access to the best practices in addition to undergoing several unnecessary interventions. The presence of a companion is associated with several beneficial practices and the reduction in some interventions, although other interventions are not impacted.

## RESUMO

**OBJETIVO:**

Analisar se a presença do acompanhante favorece a aplicação das boas práticas na atenção ao parto na região Sul do Brasil.

**MÉTODOS:**

Análise transversal do estudo longitudinal Nascer no Brasil. Foram analisados dados de 2.070 mulheres da região Sul que entraram em trabalho de parto. Os dados foram coletados entre fevereiro e agosto de 2011, por meio de entrevista e prontuário. Realizou-se análise bivariada e multivariada, calculando-se razões de prevalência brutas e ajustadas por regressão de Poisson com estimação de variância robusta. Adotou-se nível de significância de 5%.

**RESULTADOS:**

A maioria das mulheres teve o acompanhante durante o trabalho de parto (51,7%), mas poucas permaneceram com ele no parto (39,4%) ou na cesariana (34,8%). Menos da metade das mulheres teve acesso às várias práticas recomendadas, enquanto práticas não recomendadas continuam sendo realizadas. No modelo ajustado por idade, escolaridade, fonte de pagamento do parto, paridade e escore da Associação Brasileira de Institutos de Pesquisa de Mercado, a presença do acompanhante esteve estatisticamente associada à maior oferta de líquidos/alimentos (RPa = 1,34), prescrição de dieta (RPa = 1,34), uso de métodos não farmacológicos para alívio da dor (RPa = 1,37), amniotomia (RPa = 1,10), analgesia peridural ou ráqui (RPa = 1,84), adoção de posição não litotômica no parto (RPa = 1,77), permanência na mesma sala durante o trabalho de parto, parto e pós-parto (RPa = 1,62), contato pele a pele no parto (RPa = 1,81) e na cesariana (RP = 2,43), bem como redução da manobra de Kristeller (RPa = 0,67), tricotomia (RPa = 0,59) e enema (RPa = 0,49).

**CONCLUSÕES:**

Na região Sul do Brasil, além de sofrer várias intervenções desnecessárias, a maioria das mulheres não têm acesso às boas práticas. A presença do acompanhante está associada a diversas práticas benéficas e à redução de algumas intervenções, embora outras não sofram impacto.

## INTRODUCTION

The fragmented, curative, and hospital health care model characterized the construction of the Brazilian health system, privileging and consolidating individual medical practices, financed by the social security system, to the detriment of collective actions of health promotion and prevention. In this scenario, modern obstetrics and the technocratic model of labor care were easily legitimized and the complex event of delivery and birth was separated from the family and community life to become a medical and hospital matter^23^. The adoption of obstetric practices that arose with the institutionalization of labor, in order to control the physiological process of birth and rationalize work patterns, gradually characterized the parturition process as pathological and denied the intrinsic ability to give birth to the women^9^.

From a physiological, family, and social event, delivery and birth become a medical act, in which the risk of pathologies and complications becomes the rule and not the exception^23^. In this Brazilian model, practices that are considered to be harmful by the World Health Organization^30^ (WHO) and that do not have scientific evidence for their use are used, such as the routine use of a peripheral venous catheter (74.9%), zero diet (74.8%), lithotomy position (91.7%), routine use of oocytes (36.4%) and amniotomy (39.1%), Kristeller maneuver (36.1%), and excessive number of episiotomies (53.5%). However, the best practices that should be encouraged are not fully implemented, such as the use of non-pharmacological methods (NPM) for pain relief (26.7%) and the respect for the continuous presence of a companion of the woman's choice (18.8%)^10^ ^,^ ^21^. Considering that the concept of best practices in labor care is not yet described in the literature, they are understood here as the adoption of the recommendations of the WHO^30^, the implementation of scientific evidence, and the elimination of unnecessary interventions.

The Brazilian Ministry of Health has sought to qualify labor and birth care and support the implementation of scientific evidence with public health policies. One of these policies is the *Rede Cegonha* ^25^, which is a network that aims to ensure safe delivery and birth to women and newborns, including support of companions of the women's free choice.

In a systematic review of support during birth, women who receive continuous support during delivery are more likely to have spontaneous vaginal birth and less prolonged labor, they are less likely to undergo intrapartum analgesia/anesthesia, instrumental vaginal, or cesarean delivery, and they are less likely to have a baby with low fifth-minute Apgar score^17^.

In a randomized clinical trial conducted in Brazil, mothers with a companion of their choice had greater overall satisfaction with the labor and delivery experience than those in the control group. However, we need to evaluate the influence of a companion in the adoption of best practices in delivery care ^3^. In a qualitative research, women have reported that the companion provides security and the necessary emotional support, using words and gestures of care and comfort^29^ ^,^ ^31^. It has been reported that the support of a companion, associated with best practices such as showering and movement, becomes a factor capable of reducing pain and duration of labor^5^ ^,^ ^35^.

The Brazilian Ministry of Health recognizes the benefits of this practice and the publication of Law 11,108, in 2005^a^, ensures the right to a companion to the mothers during labor, delivery, and immediate postpartum within the Unified Health System (SUS), including its own network or a contracted network. Despite this, many women are still deprived of this right. General data from the research *Nascer no Brasil* suggest that Brazilian women are being exposed to the risks of iatrogenesis and the continuous presence of a companion is still not ensured for most of them (81.2%), despite being considered a marker of safety and quality of care^10^.

Thus, the objective of this study was to analyze if the presence of a companion favors the use of best practices in delivery care in the South region of Brazil.

## METHODS

This is a cross-sectional analysis of a longitudinal study, carried out from the research: *“Nascer no Brasil: inquérito nacional sobre parto e nascimento”.* That study has considered eligible all health institutions in Brazil that registered at least 500 births per year according to the Live Birth Information System (SINASC), 2007^b^. The sample were mothers hospitalized because of delivery and who had newborns at 22 gestational weeks or more, or who weighted more than 500 g at birth, with the exception of mothers whose delivery had occurred at home, in the transportation to the maternity, or in another health unit other than the one selected^20^.

Data collection was conducted in 2011 and 2012 with interviews using electronic forms. The first questionnaire was completed during interviews with the mothers within the first 24 hours after birth. The second questionnaire was filled with data available in the maternal medical records after discharge from the hospital or on the 42nd day for the woman and on the 28th day for the newborn that remained hospitalized. A total of 50 supervisors and 200 interviewers participated in the study, which makes up 27 state teams. Detailed information on data collection is described in another publication^20^.

The sample size was 90 mothers and their newborns for each health institution randomly chosen, being representative of the Brazilian regions. The sample was stratified by geographic macroregion, type of municipality (capital/interior), and hospital administration (public, mixed, or private). The sample amounted to 23,940 women, distributed in 191 municipalities in all the states of the country. The sample size in each stratum was calculated based on the cesarean rate in Brazil in 2007 of 46.6%, with a significance of 5% to detect differences of 14% (difference between mixed hospitals and private hospitals) with a power of 80%. The sample design of the *Nascer no Brasil* has been described in detail by Vasconcellos et al.^40^

For this study, we selected data from the South region of Brazil, collected between February and August 2011, in 46 health institutions randomly chosen (17 from the State of Paraná, 13 from the state of Santa Catarina, and 16 from the state of Rio Grande do Sul) amounting to 4,139 women. We included those that went into labor (spontaneous or induced), amounting to a sample of 2,070 women. We considered in labor women who had cervical dilatation of four centimeters or more.

The data used in this study emerged from two instruments: a questionnaire from the hospital interview with the mother and an instrument for collecting data from the maternal medical record.

Each obstetric practice was considered as an outcome variable. The outcome variables selected from the interview with the mother were: offering of liquid or food (yes, no), free movement (yes, no), use of NPM for pain relief (yes, no, considering bath, shower, labor ball, massage, squatting stool, rocking chair), amniotomy (yes, no), tricotomy (yes, no), enema (yes, no), non-lithotomy position (yes - lying on the side, sitting/reclining, in the bath, four supports, squatting, standing; no – lithotomy) Kristeller maneuver (yes, no), PPP room – prepartum/delivery/postpartum (yes, no), and skin-to-skin contact (yes, no). The outcome variables selected from the maternal medical report were: dietary prescription (yes, no), oxytocin prescription (yes, no), epidural or spinal analgesia (yes, no), and episiotomy (yes, no).

The two main variables of exposure were: companion in the labor and companion in the delivery, being constructed compositely, with data from the interview and the maternal medical record.

To test the association between the presence of the companion and best practices (outcomes) in labor and delivery care, multivariate models were constructed (one for each outcome), adjusted by five variables identified in the literature^21^. The adjustment variables, selected from the interview with the mother, were: age (12 to 19, 20 to 34 years, 35 or more), education level (incomplete elementary school, complete elementary school, high school, complete higher education or more), and socioeconomic score of the ABIPEME – Brazilian Association of Market Research Institutes (class A+B, class C, class D+E). The adjustment variables selected from the maternal medical records were: parity (primiparous, multiparous) and source of payment for delivery (public, private). The hypothesis studied was that the presence of a companion favors the best practices.

The economic classification criterion adopted to generate the ABIPEME variable was the one recommended by the Brazilian Association of Research Companies (ABEP), which estimates the buying power of urban individuals and families based on the ownership of goods and the education level of the head of the family^15^.

We used the statistical program Stata/SE, version 11. We applied descriptive statistics and the chi-square test used in the comparison of proportions. To estimate the crude and adjusted prevalence ratios and their respective 95% confidence intervals (95%CI), we used Poisson regression with robust variance. We considered that the variables that were part of the adjusted model belonged to the same hierarchical level. Values of p < 0.05 were considered significant.

This research is guided by Ordinance 196/96 of the National Health Council, which provides guidelines and standards for human research (CEP/ENSP - Protocol 92/10). All mothers signed the informed consent.

## RESULTS

The sociodemographic and obstetric characteristics of the participants who went into labor and, therefore, were included in this study (n = 2,070) are presented in Table 1. Most women were in the age group of 20 to 34 years (67.8%), socioeconomic class C (60.3%), multiparous (57.0%), had vaginal delivery (85.0%), with delivery in the public sector (89.7%), and remained with a companion during labor (51.7%). However, less than half had a companion during delivery (39.4%) or cesarean section (34.8%). Regarding education level, few women had complete higher education (5.7%).

**Table 1:** Sociodemographic and obstetric characteristics of women who went into labor. South Region of Brazil, 2011. (n = 2,070)

Variable		n	%	95%CI
Age (n = 2,070)				
	12–19 years	461	22.3	20.52-24.11
	20–34 years	1,404	67.8	65.78-69.81
	35 years or more	205	9.9	8.69-11.27
Education level (n = 2,064)				
	Incomplete elementary school	634	30.7	28.76-32.74
	Complete elementary school	652	31.6	29.62-33.63
	Complete high school	661	32.0	30.04-34.07
	Complete higher education or more	117	5.7	4.75-6.75
ABIPEME score (n = 2,057)				
	A+B	576	28.0	26.10-29.98
	C	1,240	60.3	58.15-62.38
	D+E	241	11.7	10.39-13.18
Parity (n = 2,069)				
	Primiparous	890	43.0	40.89-45.16
	Multiparous	1,179	57.0	54.84-59.10
Type of delivery (n = 2,070)				
	Vaginal	1,760	85.0	83.42-86.50
	Cesarean	310	15.0	13.50-16.58
Source of payment / delivery (n = 2,070)				
	Public	1,857	89.7	88.32-90.95
	Private	213	10.3	9.05-11.67
Presence of companion				
	In labor (n = 1,975)	1,021	51.7	49.49-53.90
	In the delivery (n = 1,759)	693	39.4	37.14-41.70
	In the cesarean (n = 310)	108	34.8	29.71-40.34
Labor practices				
	Diet offer (n = 1,938)	354	18.2	16.57-20.01
	Dietary prescription (n = 1,374)	463	32.7	30.30-35.19
	Free movement (n = 1.893)	1,124	59.2	57.02-61.44
	NPM pain relief (n = 1,975)	676	32.7	30.67-34.71
	Oxytocin (n = 1,875)	1,008	52.2	49.97-54.42
	Amniotomy (n = 1,858)	954	51.2	48.96-53.50
	Trichotomy (n = 1,875)	843	43.6	41.46-45.88
	Enema (n = 1,875)	714	37.0	34.85-39.15
	Labor analgesia (n = 1,875)	175	9.1	7.86-10.43
Practices in the delivery				
	Non-lithotomy position	90	5.1	4.19-6.27
	Episiotomy	912	44.1	41.93-46.21
	Kristeller maneuver	571	27.6	25.76-29.62
	PPP room	289	16.4	14.77-18.25
	Skin-to-skin contact	889	43.3	41.21-45.50

ABIPEME: Economic classification recommended by the Brazilian Association of Research Companies; NPM: non-pharmacological method; PPP: prepartum, delivery, and postpartum

Regarding obstetric practices performed during labor, less than half of the women were allowed to drink or eat during this period (32.7%). Most of them were able to move (59.2%), but few used a NPM for pain relief (32.7%) – bathtub, shower, labor ball, massage, squatting stool, rocking chair. Most received oxytocin (52.2%) and had their membranes ruptured artificially (51.2%). Most of them had trichotomy (43.6%) and enema (37.0%), and few received epidural or spinal anesthesia (9.1%). During delivery, very few of them could choose a different lithotomy position to give birth (5.1%), and many received an episiotomy (44.1%) or uterine fundal pressure to push the baby (Kristeller maneuver) (27.6%). Few women remained in the same room during prepartum, delivery, and postpartum (PPP room) (16.4%) and less than half had skin-to-skin contact with the baby soon after birth (43.3%).

Table 2 presents the crude and adjusted prevalence and prevalence ratios of the obstetric practices performed during labor, according to the presence of a companion. The presence of a companion during labor, after adjusting for the variables of age, education level, source of payment of delivery, parity, and ABIPEME score, was significantly associated with a greater supply of liquid or food (aPR = 1.34, 95%CI 1.10–1.63), prescription of some type of diet (aPR = 1.34, 95%CI 1.15–1.57), use of NPM for pain relief (aPR = 1.37, 95%CI 1.21–1.56), and use of amniotomy (aPR = 1.10, 95%CI 1.01–1.21) and epidural or spinal analgesia (aPR = 1.84, 95%CI 1.33–2.54). In addition, the presence of a companion during labor was associated with reduced use of trichotomy (aPR = 0.59, 95%CI 0.53–0.65) and enema (aPR = 0.49, 95%CI 0.43–0.56).

**Table 2:** Obstetric practices performed during labor, according to the presence of companion. South Region of Brazil, 2011. (n = 2,070)

Variable	Presence of companion in labor							
	Yes		No		PR	95%CI	aPR^c^	95%CI
	n	%	n	%				
Diet offer (n = 1,938)	215	21.1	139	15.1^a^	1.39	1.15—1.69”	1.34	1.10-1.63^b^
Dietary prescription (n = 1,3 74)	2 75	37.5	177	27.7^a^	1.35	1.16—1.58”	1.34	1.15-1.57^b^
Free movement (n = 1.893)	614	62.0	509	56.4^a^	1.10	1.02-1.19^a^	1.07	0.99-1.15
NPM pain relief (n = 1,975)	411	40.2	265	2 7.8^a^	1.45	1.28-1.64	1.37	1.21-1.56^b^
Oxytocin (n = 1,875)	524	54.1	461	50.8	1.06	0.98-1.16	1.05	0.96-1.15
Amniotomy (n = 1,858)	517	53.5	435	48.8^a^	1.09	1.00-1.20”	1.10	1.01-1.21^b^
Trichotomy (n = 1,875)	321	33.2	503	55.5^a^	0.60	0.54-0.66^a^	0.59	0.53-0.65^b^
Enema (n = 1,875)	247	25.5	453	49.9^a^	0.51	0.45-0.58^a^	0.49	0.43-0.56^b^
Labor analgesia (n = 1,875)	117	12.1	46	5.1^a^	2.38	1.71-3.31^a^	1.84	1.33-2.54^b^
Vaginal delivery (n = 1,975)	889	87.1	853	89.4	0.97	0.94-1.00	0.99	0.96-1.02

PR: prevalence ratio; aPR: adjusted prevalence ratio; NPM: non-pharmacological method; ABIPEME: Economic classification recommended by the Brazilian Association of Research Companies

a Chi-square test: p < 0.05

b Wald test: p < 0.05

c Adjusted for age, education level, payment of the delivery (public or private), parity, and ABIPEME score.

Table 3 presents the crude and adjusted prevalence and prevalence ratios of the obstetric practices performed during labor, according to the presence of a companion during delivery/birth. During delivery, the presence of a companion remained significantly associated with the adoption of different lithotomy positions by the woman (PR = 1.77, 95%CI 1.16–2.72), stay in the PPP room (PR = 1.62, 95%CI 1.31–2.00), and skin-to-skin contact between the mother and the baby soon after birth, both in the delivery (PR = 0.81, 95%CI 1.64–1.99) and cesarean section (PR = 2.43, 95%CI 1.22–4.85). In addition, the Kristeller maneuver was significantly less performed in women who were with a companion (PR = 0.67, 95%CI 0.58–0.78).

**Table 3:** Obstetric practices performed during delivery, according to the presence of companion at that time, in women who went into labor. South Region of Brazil, 2011. (n = 2,324)

Variable		Presence of companion in the delivery							
		Yes		No		PR	95%CI	aPR^c^	95%CI
		n	%	n	%				
In the delivery									
	Non-lithotomy position (n = 1,753)	49	7.1	41	3.9^a^	1.83	1.22-2.74”	1.77	1.16-2.72^b^
	Episiotomy (n = 1,759)	393	56.7	519	48.7^a^	1.16	1.06-1.2 7”	1.01	0.93-1.11
	Kristeller maneuver (n = 1,754)	188	27.2	383	36.0^a^	0.75	0.65-0.87^a^	0.67	0.58-0.78^b^
	PPP room (n = 1,757)	133	19.2	156	14.7”	1.31	1.06-1.62”	1.62	1.31-2.00^b^
	Skin-to-skin contact (n = 1,741)	456	66.1	390	3 7.1^a^	1.78	1.62-1.96”	1.81	1.64-1.99^b^
In the cesarean									
	Skin-to-skin contact (n = 309)	27	25.0	16	8.0^a^	3.14	1.77-5.57”	2.43	1.22-4.85^b^

PR: prevalence ratio; aPR: adjusted prevalence ratio; PPP: prepartum, delivery, postpartum; ABIPEME: Economic classification recommended by the Brazilian Association of Research Companies

a Chi-square test: p < 0.05

b Wald test: p < 0.05

c Adjusted for age, education level, payment of the delivery (public or private), parity, and ABIPEME score.

The frequency of some interventions that are considered unnecessary or inappropriate, such as episiotomy and oxytocin, were not significantly affected by the presence of a companion.

## DISCUSSION

The results show that most women in the South region do not have access to the best practices in the birth and delivery care. The presence of a companion was more frequent in labor than in delivery or cesarean section. We identified high rates of enema, trichotomy, Kristeller maneuver, amniotomy, oxytocin, episiotomy, and water and food restriction. Moreover, little more than half of the mothers had freedom of position and movement and very few managed to give birth in a non-lithonomy position. Most did not have access to the NPM for pain relief, PPP rooms, and skin-to-skin contact with the newborn soon after delivery. However, the presence of a companion in the labor implied a greater supply of liquids or food, dietary prescription, NPM for pain relief, and reduced trichotomy and enema. In the delivery, the presence of a companion was associated with the adoption of a non-lithonomy position, PPP room, skin-to-skin contact, and reduced use of the Kristeller maneuver.

The fact that companions are more present during labor than in the delivery is similar to the findings of a study carried out in the State of Santa Catarina, Brazil, in which the stay of a companion in the delivery room was not allowed in the same proportion as in the other obstetrical service sites^5^. This restriction may be because many professionals do not consider delivery/birth as a family event, but rather a medical act, which should be cared for in a “sterile” environment, without the presence of laypersons, because of their potential for risk and the possibility of interventions in the event of complications.

Trichotomy and enema, performed in almost half of the women, are harmful and ineffective practices that should be discouraged and eliminated because they do not have a significant beneficial effect on the rates of infection or dehiscence of the perineal wound or other neonatal infections^2^.

The Kristeller maneuver was widely used, despite the lack of sufficient evidence to support its recommendation, being similar to other Brazilian studies^11^ ^,^ ^21^. In addition to providing greater maternal discomfort, this maneuver brings deleterious effects to the uterus, perineum, and fetus and it is ineffective in reducing the second stage of labor^1^ ^,^ ^24^.

Although delivery is a physiological event, other interventions, such as routine oxytocin infusion and amniotomy, have been performed to prevent prolonged labor. In this study, half of the women received at least one of the two interventions, even though there was insufficient evidence to justify these practices^33^.

In addition to the risks associated with the liberal use of oxytocin for the correction of the dynamics during labor, including maternal exhaustion, uterine hyperstimulation, rupture of the uterus or placenta, and fetal distress^22^, continuous intravenous infusion also limits the freedom of movement of the women, which may prolong the duration of labor. In relation to early amniotomy, a review study has concluded that this procedure should not be routinely recommended and it should be a decision discussed with the women before using it^36^.

The high prevalence of episiotomy in Latin America^6^ was also found here. Episiotomy is essentially characterized as a second-degree laceration, capable of breaking down several muscle groups and increasing the occurrence of third and fourth degree lacerations^38^. Ideally, the rate of episiotomy should not exceed 10%, a goal considered reasonable by the WHO^30^. Several countries have pursued a restrictive policy for episiotomy. In the USA, the percentage dropped significantly from 17.3% in 2006 to 11.6% in 2012^13^. However, high rates are still prevalent; in a study carried out in Spain, the rate was 33.5% in 2011^39^, and in a research conducted in England, the rate was 25%^32^.

Few of the women studied received some type of diet. This may be related to the resistance of health professionals in relation to water and food intake by the mother, although scientific evidence shows deleterious effects of fasting on labor^c^ and the WHO considers the provision of oral fluids a useful practice that should be stimulated^30^.

Almost half of the women studied did not move during labor, despite movement and vertical positions reducing the pain and duration of that period^19^.

In addition to these various interventions, we highlight that most women did not have access to NPM for pain relief, despite the efficacy of these methods^7^. These results differ from other studies with smaller samples, performed in public hospitals in the South region of Brazil, in which women had more access to NPM, indicating that these experiences are still specific and depend on the care philosophy^5^ ^,^ ^16^.

The PPP system and skin-to-skin contact in the delivery room occurred in less than half of the mothers. The PPP room allows the presence of persons of the mother's choice and a humanized and safe care for the mother-baby binomial^37^. Early skin-to-skin contact provides immediate and long-term benefits as it improves the effectiveness of the first feeding, regulates the body temperature of the baby, and contributes to maternal attachment^27^.

In this highly interventionist reality, the presence of a companion was associated with a reduction in the use of unnecessary or inadequately used interventions, either in labor, such as the use of amniotomy, trichotomy, and enema, or in the delivery, such as reducing the use of Kristeller maneuver. These practices were not evaluated in the systematic review updated in 2013^17^.

In addition, the presence of a companion during labor favored the supply of liquids and food and the use of NPM for pain relief. In the delivery, the companion favored the adoption of a non-lithonomy position, the care of the woman in the PPP system, and skin-to-skin contact soon after birth. In women who underwent cesarean section, early skin-to-skin contact was 2.4 times more likely when a companion was present. The implementation of these best practices, supported by scientific evidence, was also not evaluated in the systematic review on the support at birth^17^.

These findings may be related to the care philosophy implemented in health institutions, since, by allowing a companion, other practices are also recognized as beneficial and become part of the routine, as already mentioned in other research studies that portray local experiences in Brazil^5^ ^,^ ^18^. In addition, when a companion is welcomed and encouraged to perform actions of physical comfort, he participates in the application of NPM^16^ and acts as intermediation, negotiating the woman's wishes with the health team^17^.

In Brazil, health institutions that allow the presence of a companion are those that seek to reduce unnecessary interventions, which have no evidence and are not recommended by the WHO for delivery care, and those that have implemented minimal changes in the environment and in the furniture, such as having chairs for all the companions^5^ ^,^ ^9^ ^,^ ^10^. Thus, the correlation between the presence of a companion and the adoption of best practices in institutions may have contributed to these results. We should also consider that the health professional tends to change his or her attitude when a companion is present, which can be observed in studies that show that women are more satisfied with the experience of delivery and with the guidelines and care received from health professionals when they have a companion of their choice^3^ ^,^ ^4^ ^,^ ^28^.

Despite the benefits presented, after adjusting for the control variables, the presence of a companion in the South region did not have any impact on free movement, type of delivery, and rates of episiotomy and oxytocin.

The presence of a companion may not have reduced the prescription of oxytocin and episiotomy because they are routinely used in many institutions, often without the knowledge or authorization of the mothers, further reinforcing a technocratic and mechanistic model of delivery care. These findings are similar to those found in a systematic review^17^, in which the support had no impact on the occurrence of oxytocin and episiotomy. In a qualitative study, women who had an episiotomy were unaware of the purpose of the procedure and reported that they were not asked to authorize the procedure^14^.

International studies seek to identify the reasons that hinder the appropriation of evidence-based interventions and the shift in care practice around the world, including practices that could be used in places with few resources. Among the reasons, the lack of access of professionals to scientific knowledge in developing countries stands out, as well as the lack of interest of professionals and managers of health services in the production of better obstetric and neonatal care^8^ ^,^ ^12^ ^,^ ^34^.

In this study, epidural or spinal analgesia was more frequent in women with a companion. This result differs from that found in a systematic review, in which the support reduced the need for analgesia^17^. Although this procedure interferes with the evolution of labor, the result of this study may be related to the reversal of the concept present in the Brazilian obstetric reality, in which the excess of interventions is often considered a synonym of good care.

It is important to note that most of the variables analyzed here were selected from the interview questionnaire used with the mother, being self-reported and therefore susceptible to understanding bias.

Another limitation is related to the study design, that is, because this is a cross-sectional study, we only estimated the association of the presence of a companion with best practices in delivery care, but we cannot indicate causality. Thus, we need to consider that the existing association may be due to the care model adopted in some institutions, in which the presence of a companion is included in a list of best practices already performed. The *Diretriz Nacional de Assistência ao Parto Normal,* published in 2016, highlights that the continued support from the companion of the women's choice does not waive the support provided by the hospital staff from the adoption of the best practices available^26^.

The continuous support provided by a companion during labor/delivery is characterized as a protective factor as it favors the reduction of interventions and harmful and aggressive practices at a time when the mother and the newborn are extremely vulnerable to the hospital routines and the decisions of professionals. In this way, the adaptation of maternity hospitals to include and accommodate a companion is imperative. Regarding the use of resources needed to ensure continued support, we highlight that the support from the companion of the woman's choice and her own social network, based on the benefits already described, could favor resource saving in relation to the number of professionals needed to ensure this permanent support^26^, which could be destined to the structural adequacy of institutions to the presence of a companion.

In order to ensure the presence of a companion of the woman's free choice, responsible agencies need to supervise health institutions linked to the SUS or supplementary health, and changes need to happen in the training of professionals to assist the delivery. In the current debate on public policies on delivery care, this right needs to ensure, which has historically been the agenda of the womens movement.

Women should also be encouraged, in social networks and organized groups, to strive for a dignified and humanized care, which rescues the feminine protagonism and see the delivery as a physiological and family event.

Considering the Brazilian context, in which the companions of the woman's choice have been considered as the support provider, further studies are needed to explore other results from their presence, increasing the knowledge about their role in delivery and birth.

## Footnotes

a Presidência da República (BR), Casa Civil, Subchefia para Assuntos Jurídicos. Lei N° 11.108, de 7 de abril de 2005. Altera a Lei n° 8.080, de 19 de setembro de 1990, para garantir às parturientes o direito à presença de acompanhante durante o trabalho de parto, parto e pós-parto imediato, no âmbito do Sistema Único de Saúde - SUS. Brasília (DF); 2005 [cited 2017 Jun 16]. Available from: http://www.planalto.gov.br/ccivil_03/_ato2004-2006/2005/lei/l11108.htm

b Ministério da Saúde (BR). Sistema de Informação da Atenção Básica -SIAB. Brasília (DF); 2008 [cited 2017 Jun 16]. Available from: http://www2.datasus.gov.br/SIAB/index.php?area=04A01&item=1

c Amorim M. Assistência ao parto baseada em evidências. Rio de Janeiro; 2012 [cited 2017 Jun 16]. Available from: http://estudamelania.blogspot.com.br/2012/09/assistencia-ao-parto-baseada-em.html
